# Using simulation to increase resident comfort discussing social determinants of health

**DOI:** 10.1186/s12909-021-03044-5

**Published:** 2021-12-06

**Authors:** John M Morrison, Sarah M. Marsicek, Akshata M Hopkins, Robert A Dudas, Kimberly R Collins

**Affiliations:** 1 Department of Pediatrics, Johns Hopkins University School of Medicine, MD Baltimore, USA; 2 Division of Pediatric Hospital Medicine, Department of Pediatric Medicine, Johns Hopkins All Children’s Hospital, FL St Petersburg, USA; 3 Division of Pediatric Hospital Medicine, Department of Pediatric Medicine, AdventHealth for Children, FL Orlando, USA

**Keywords:** Social determinants of health, Simulation, Graduate medical education

## Abstract

**Background:**

Social determinants of health (SDoH) play an important role in pediatric health outcomes. Trainees receive little to no training on how to identify, discuss and counsel families in a clinical setting. The aim of this study was to determine if a simulation-based SDoH training activity would improve pediatric resident comfort with these skills.

**Methods:**

We performed a prospective study of a curricular intervention involving simulation cases utilizing standardized patients focused on four social determinants (food insecurity, housing insecurity, barriers to accessing care, and adverse childhood experiences [ACEs]). Residents reported confidence levels with discussing each SDoH and satisfaction with the activity in a retrospective pre-post survey with five-point Likert style questions. Select residents were surveyed again 9–12 months after participation.

**Results:**

85% (33/39) of residents expressed satisfaction with the simulation activity. More residents expressed comfort discussing each SDoH after the activity (Δ% 38–47%; all *p* < .05), with the greatest effect noted in post-graduate-year-1 (PGY-1) participants. Improvements in comfort were sustained longitudinally during the academic year. More PGY-1 participants reported engaging in ≥ 2 conversations in a clinical setting related to food insecurity (43% vs. 5%; *p* = .04) and ACEs (71% vs. 20%; *p* = .02).

**Discussion:**

Simulation led to an increased resident comfort with discussing SDoH in a clinical setting. The greatest benefit from such a curriculum is likely realized early in training. Future efforts should investigate if exposure to the simulations and increased comfort level with each topic correlate with increased likelihood to engage in these conversations in the clinical setting.

**Supplementary Information:**

The online version contains supplementary material available at 10.1186/s12909-021-03044-5.

## Introduction

Child health is directly influenced by social determinants, or the circumstances in which patients and their families live and work [1, 2]. Children living in poverty have higher rates of developmental and behavioral concerns [﻿^2^] and housing insecurity is associated with diarrheal illness, asthma, frequent ear infections and lead poisoning [3]. In addition to poverty, children exposed to toxic stress, defined as frequent or chronic activation of the stress response due to repeated exposure to significant stressors, are at an increased risk of developing chronic diseases and substance abuse disorders as adults [4]. Given the effects of these SDoH, and others, on families, the American Academy of Pediatrics Policy Statement on Poverty and Child Health in the United States recommends pediatricians screen for risk factors within social determinants of health (SDoH)﻿ and provide a medical home addressing the needs of families that screen positive [5]. There is evidence that doing so can result in improved family connectivity with community resources [2].

Historically, pediatric trainees have inadequate training to identify and intervene on the unmet needs associated with SDoH [6]. Recent educational interventions focused on improving the knowledge of and attitudes towards addressing SDoH in a clinical setting have shown promising results [7, 8]. Didactic- and immersion-based efforts to provide training to pediatric residents on SDoH have resulted in improved trainee knowledge and comfort with discussing social determinants of health [9]. Simulation-based educational experiences targeted at communicating during difficult patient encounters, such as those involving SDoH, are desirable among pediatric residents and may improve comfort with such encounters more than didactics and immersion experiences alone [10]. Training using simulation has been widely used for both teaching and assessment (including procedural skills training, team training for response to medical emergencies and communication skills training) for physician trainees and other areas of health professions education [11–13]. However, outcomes associated with simulation exercises on addressing trainee comfort with discussing SDoH with either standardized patients or actual patients have not been widely reported. An important first step in exploring the potential for simulation to augment training in screening for and discussing SDoH patients is to first determine if simulation regarding this topic is feasible and whether or not it can improve lower level outcomes. Informed by our institution’s community health needs assessment, we chose four of the many SDoH around which to develop simulation cases for pediatrics residents [14]. These cases focused on addressing food insecurity, housing insecurity, barriers to accessing health care, and adverse childhood experiences ( ACEs) utilizing simulated parents. We hypothesized that simulation-based training would improve resident self-reported comfort with discussing SDoH in a clinical setting.

## Methods

### Study design

This was a prospective mixed-methods study of a curricular intervention conducted within a single pediatric residency program. We selected a pre-post quasi-experimental design to evaluate outcomes. Participants consisted of two cohorts of pediatric residents separated by one year. Residents from the first cohort were re-surveyed 9–12 months after participating in the initial simulation. This study and its procedures were approved by the Johns Hopkins University Institutional Review Board and all research activities were conducted in concordance with the United States Department of Health and Human Services Common Rule for human subjects research. All participants provided informed written consent for participation in this study.

### Setting

**Fig. 1:**
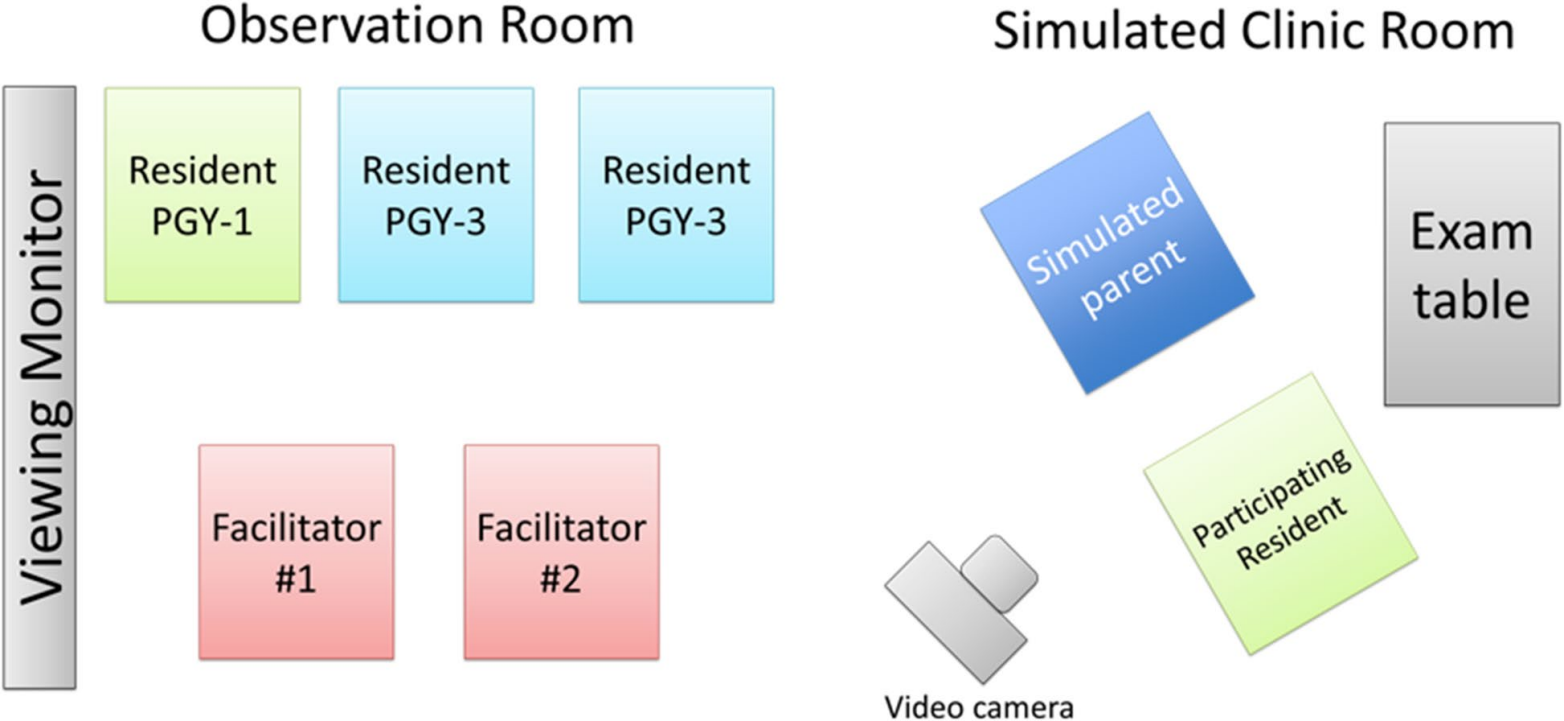
Schematic of simulation setup. The participating resident interacted with the simulated parent actor in a clinical room within the simulation center. This interaction was recorded via video camera and transmitted to an observation room where other residents from the group and the facilitators could directly observe on a viewing monitor. Upon completion of the scenario, the participating resident would then join the group in the observation room for debriefing

The curriculum was implemented at the Johns Hopkins All Children’s Hospital Center for Simulation and Innovative Education. The simulated clinic rooms contained audio and video equipment transmitting to a second room in which a large monitor and speaker system allowed live viewing of the simulated clinic encounter (Fig. 1).

### Simulated Parents

Simulated parents (SPs) who had previous training and experience in healthcare simulation played the role of the caregiver. Weeks in advance, SPs received the scenario outlining each case’s learning objectives, basic patient information, resident learner description, and SP role description (including priming on when to reveal pertinent information in response to specific resident prompts). Each SP was oriented to the simulation center and allowed to ask questions prior to the simulation session. No actors were used to play the role of the child, and the simulated discussions between participants and caregivers were assumed to take place without the child’s presence.

### Participants

Our institution’s residency program consists of 36 total pediatric residents with 12 residents per Post-Graduate-Year (PGY). Two cohorts of PGY-1 and PGY-3 residents participated in all simulation experiences as part of a required didactic and experiential curriculum teaching on cultural competency, implicit biases, and health inequity in the fall of 2018 and 2019 [15]. Of note, PGY-3 residents from the first cohort had already received a 45-minute didactic-based training on the recognition and effects of ACEs on child health as part of a project targeting screening for ACEs in the outpatient clinic setting [16].

### Scenarios

 We designed four separate clinical scenarios in which SPs role-played the caregiver of a child affected by specific SDoH. For each scenario, residents initiated contact with the SP, disclosed relevant screening tool results, and identified resources for the family relevant to each scenario. Residents and SPs were instructed to address the relevant SDoH and ignore other tangential medical discussions unless felt related to the SDoH. After the encounter, participating residents reunited with their peers and facilitators for a debriefing session. Each scenario lasts approximately 20 min including the debrief. Trainees reviewed information on community resources pertaining to each SDoH 24 h prior to the simulations.

#### Scenario 1: Food insecurity

This scenario takes place in the context of a well-child visit. The only medical concern identified during the visit was that the child was overweight. Residents were provided the results of a validated two-item food insecurity screening tool that indicated the child was at-risk for living in a food insecure home [17]. The resident physician was then tasked with introducing the results of the screening tool, assessing for any needs as it pertained to the family, and pointing the family to additional resources.

#### Scenario 2: Housing insecurity

This scenario takes place during a well-care visit for a child with persistent asthma. The participant was given information that the child has persistent wheezing and is needing to use short-acting β-agonist medication frequently despite using a daily inhaled corticosteroid and long-acting β-agonist combination medication. As part of the priming for the scenario, the resident was given the results of the Accountable Health Communities Core Health-Related Social Needs Screening Questions indicating a family was at-risk for housing insecurity [18]. The resident physician was then tasked with introducing the results of the screening tool, assessing for any needs as it pertained to the family, and pointing the family to additional resources.

#### Scenario 3: Limited access to health care

This scenario takes place during a follow-up visit for a child with epilepsy after recently being discharged from the hospital for increasing seizure activity. During that hospitalization, a serum level of the patient’s anti-epileptic medication was sub-therapeutic. The participant was primed with information that the patient had missed multiple clinic appointments in the preceding six months. The resident physician was then tasked with discussing reasons for the sub-therapeutic medication level and was primed to pursue avenues pertaining to medication non-compliance.

#### Scenario 4: Exposure to ACEs

This scenario takes place during a well-child visit. The patient’s legal guardian, a grandparent, reported concerns regarding the child’s symptoms of anxiety and difficulty sleeping. The participant was primed with information obtained from a standardized screening tool to assess a child’s exposure to adverse childhood experiences and other stressors [19]. Results from this tool indicated that the child had been exposed to a five different ACEs. The resident physician was then tasked with discussing reasons for the child’s symptoms in the context of the screening results.

### Implementation

Participants were separated into groups of four (two PGY-1 and two PGY-3) for the simulations. We purposefully used this approach to promote the opportunity for near-peer feedback, coaching, role-modeling and teaching. During the course of this 90-minute session, PGY-1 and PGY-3 residents each participated in one of the four scenarios with the task of addressing SDoH in a clinical setting and recommending initial steps to address unmet social needs. Participants directly observed the remaining three scenarios performed by peer residents. PGY-1 residents served as the providers in the food or housing insecurity scenarios; PGY-3 residents served as the providers in the limited access to health care and exposure to ACEs scenarios. Residents not serving as the primary care provider observed the simulation in the satellite room of the simulation center (Fig. 1). At the conclusion of the scenario, a debrief session was led by faculty members with experience facilitating simulation debriefs, using debriefing scripts based on advocacy-inquiry techniques [20]. Simulated caregivers also participated in the debrief session. Relevant local community resources pertaining to each scenario were included in the debrief discussions where appropriate.

### Evaluation framework

Evaluation of our program was based on the first three levels of Kirkpatrick’s model of reaction, learning, behavior and results [21, 22].

#### Level 1: Participation/Reaction

Residents were asked to complete an evaluation at the conclusion of each session. Our survey instrument included two questions about the educational value of the experience. In addition, a single open-ended question allowed residents to provide recommendations to improve the sessions.

#### Level 2: Learning

At the conclusion of all four scenarios, resident comfort level was assessed using paper-based retrospective pretest posttest surveys (see Supplemental Fig. 1) [23]. Residents responded to each statement using a parametric Likert-type scale ranging from “Disagree Strongly” to “Agree Strongly”. All responses were anonymous and reviewed in aggregate. PGY-1 and PGY-3 residents from the first cohort completed a follow-up survey 9–12 months later to assess for retention of comfort with discussing each scenario.

#### Level 3: Behavioral Change

Residents participating in the follow-up survey were asked to “identify one thing they did differently in continuity clinic” as a result of participation in the simulation sessions. The continuity clinic is a primary care clinic in which residents provide general pediatric care to patients on average one half-day per week throughout their residency training. Additionally, residents provided an estimate of their number of conversations in continuity clinic with each of the four SDoH scenarios before the simulation (and after, if applicable) using categorical responses of 0, 1, 2–5, or > 5 conversations.

### Statistical analysis

The primary outcome variable of interest was the change in the proportion of trainees reporting comfort with addressing the SDoH scenarios. The proportion of residents expressing agreement with each statement (characterized as “Agreement” or “Strong Agreement”) were categorized by level of training as well as for the entire study cohort. We also reported the frequency of resident categorical responses estimating the number of times they had discussed each of the four SDoH scenarios in the resident continuity clinic. For statistical analysis, frequencies were compared using a two-tailed Fisher exact test utilizing a significance level of 0.05.

## Results

A total of 48 pediatric residents participated in the simulation exercise (24 per academic year) and 24 residents from the first year of the study were approached for longitudinal questioning. The overall survey response rate of residents opting to have their surveys included in this study was 81% (39/48) for the immediate post-exercise survey and 54% (13/24) for the longitudinal follow-up survey. 83% (20/24) of PGY-1 and 79% (19/24) of PGY-3 trainees were included in the immediate post-exercise survey. 58% (7/12) of PGY-1 and 50% (6/12) of PGY-3 trainees were included in the longitudinal survey.

### Experience with social determinants of health

**Fig. 2:**
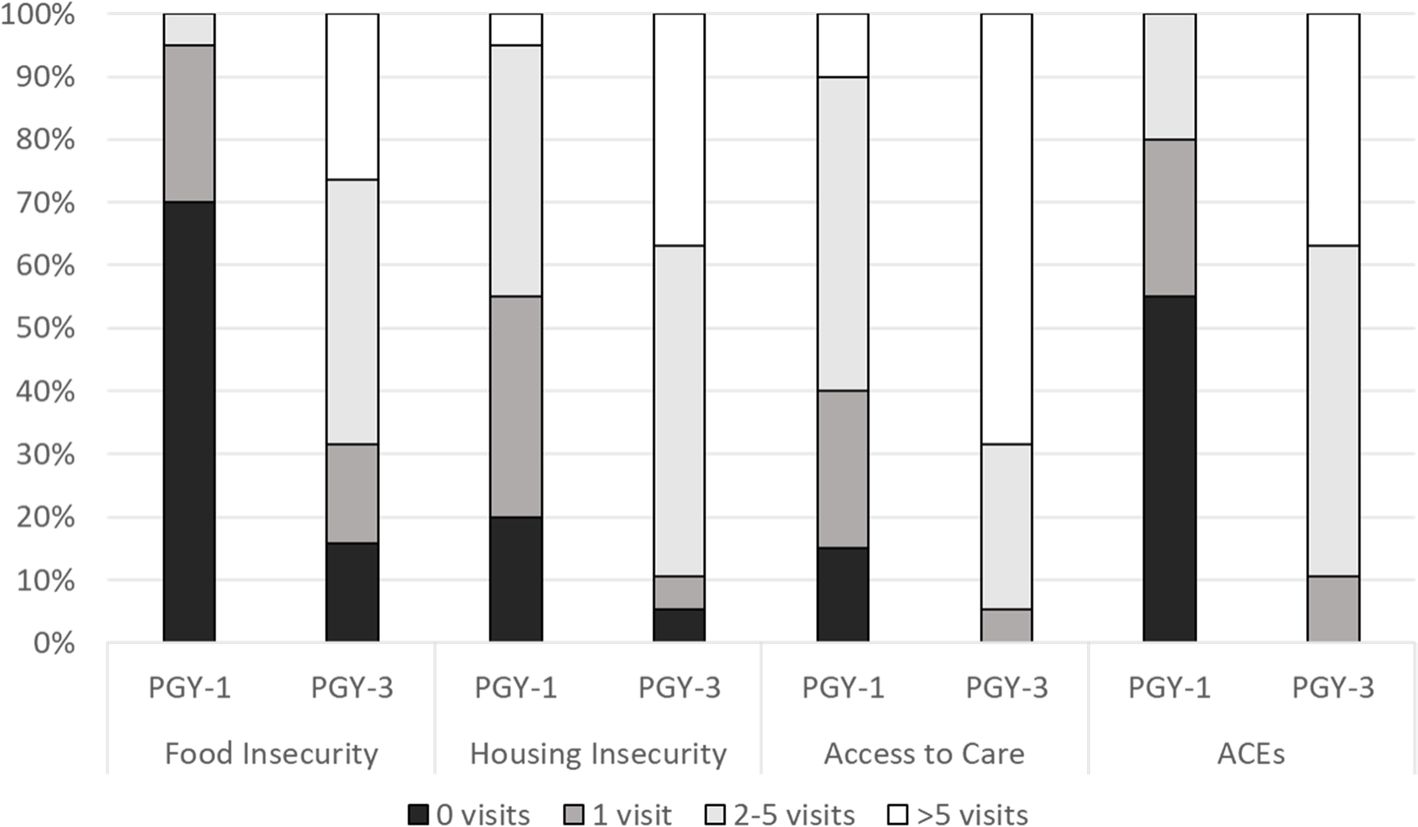
Participant self-reported clinical experience related to each SDoH scenario

Results of resident self-reported experience with each SDoH prior to the simulation exercise are listed in Fig. 2. Many PGY-1 residents reported little experience discussing each SDoH prior to the activity, with more than half of participants stating they had < 2 clinical conversations about food insecurity (92%), unsafe housing environment (67%) and adverse childhood experiences (92%) prior to the simulation exercise. More PGY-3 residents reported ≥ 2 experiences with all SDoH scenarios compared to PGY-1 residents except for the limited access to healthcare scenario (Fig. 2).

### Level 1: Participation and reaction to exercise

Overall, residents expressed satisfaction with the activity, with 85% (33/39) agreeing that they would recommend or strongly recommend offering this simulation in the future and that the simulation was a valuable use of their time. Among the cohort of residents that completed the longitudinal follow-up survey, 100% (13/13) continued to agree or strongly agree that the session was valuable and that it should be offered to trainees in the future. A summary of select qualitative comments from participants are included in Table 1. In general, residents reported learning communication skills relevant to discussing SDoH and appreciated that there are a variety of approaches to addressing SDoH in a clinical setting. Resident participants also acknowledged that not all issues related to SDoH can be immediately addressed in a single clinic visit.

**Table 1:** Select resident responses

**List one thing you learned during this session:**
“How to normalize discussions of difficult situations”
“Avoid early search satisfaction”
“It’s okay to probe patients to get difficult answers. Just check your own biases/emotions and not be offended if they don’t open up”
“The importance of pausing for reflection”
“It’s okay not to solve a problem sometimes. It is important to ask questions even if you don’t immediately have an answer”
**How could this session be improved?**
“ Have the standardized patients be a bit more reserved in letting out information for PGY-1s”
“It would be useful to have more guidance on the resources we have. The prompts were lengthy-it may be useful to give them to everyone before starting session to avoid delays”
“I liked the 1st /3rd year combo”
“Sims are always stressful, but I got great feedback

### Level 2: Learning

Overall, a greater proportion of residents agreed they were comfortable with discussing each SDoH (*p* < .05 for all scenarios; Table 2). This effect was largest for PGY-1 participants for whom only 5–25% reported comfort with each conversation prior to the scenario, whereas 68–75% reported comfort after the simulation exercises (*p* < .01 for all scenarios). More PGY-3 participants reported pre-activity comfort overall compared to PGY-1 participants (58–100%). Only the proportion of residents reporting comfort after the unsafe housing scenario (95%; 18/19) compared to before the scenario (68%; 13/19) was significantly different (*p* = .04). These improvements were maintained longitudinally over the course of the academic year (Table 3). No significant differences were detected in the proportion of respondents expressing comfort with each SDoH scenario at the end of the simulation exercise and 9–12 months after completing the exercise.

**Table 2:** Proportion of residents expressing agreement with having confidence discussing social determinant of health

	PGY-1 (n = 20)			PGY-3 (n = 19)			Total (n = 39)		
Social Determinant	Pre	Post	*p*	Pre	Post	*p*	Pre	Post	*p*
Food insecurity n, (%)	5 (25)	15 (75)	< .01	14 (74)	19 (100)	.05	19 (49)	34 (87)	< .01
Unsafe housing environment n, (%)	2 (10)	15 (75)	< .01	13 (68)	18 (95)	.04	15 (38)	33 (85)	< .01
Access to care n, (%)	1 (5)	14 (70)	< .01	11 (58)	16 (84)	.07	12 (31)	30 (77)	< .01
Adverse childhood experiences n, (%)	1 (5)	13 (65)	< .01	12 (63)	15 (79)	.48	13 (33)	28 (72)	.04

**Table 3:** Proportion of residents expressing agreement with having confidence discussing social determinants of health measured at the end of the simulation session and 9–12 months later

	PGY-1			PGY-3			Total		
Social Determinant	End of Session (n = 12)	9–12 mos. Later (n = 7)	*p*	End of Session (n = 12)	9–12 mos. Later (n = 6)	*p*	End of Session (n = 24)	9–12 mos. Later (n = 13)	*p*
Food insecurity n, (%)	5 (42)	6 (86)	.15	12 (100)	6 (100)	1.0	19 (79)	12 (92)	.39
Unsafe housing environment n, (%)	7 (58)	7 (100)	.11	11 (92)	6 (100)	1.0	18 (63)	13 (100)	.07
Access to care n, (%)	8 (67)	4 (57)	1.0	10 (83)	5 (83)	1.0	18 (75)	9 (69)	1.0
Adverse childhood experiences n, (%)	6 (50)	2 (29)	.63	9 (75)	6 (100)	.52	15 (63)	8 (62)	1.0

### Level 3: Behavioral Changes

The proportion of participants reporting conversations relating to each SDoH at the time of the simulation (n = 39) and at the longitudinal follow-up (n = 13) are displayed in Table 4. Among PGY-1 participants that completed the longitudinal follow-up survey, the percentage of residents reporting ≥ 2 clinical conversations for each SDoH increased (Δ%) by 38–51%, although only the Δ% for ACEs experiences was significant (51%, *p* = .02). PGY-3 residents reported Δ% ranging from − 28 to 15%, however none of these reached significance. Residents completing the follow-up survey reported several behavioral changes as the result of the simulation exercise (Table 5).

**Table 4:** Longitudinal self-reported conversations of two or more encounters for each social determinant of health

	PGY-1			PGY-3			Total		
Social Determinant	End of Session (n = 20)	9–12 mos. Later (n = 7)	*p*	End of Session (n = 19)	9–12 mos. Later (n = 6)	*p*	End of Session (n = 39)	9–12 mos. Later (n = 13)	*p*
Food insecurity n, (%)	1 (5)	3 (43)	.04	13 (68)	5 (83)	.64	14 (36)	8 (62)	.51
Unsafe housing environment n, (%)	9 (45)	6 (86)	.09	17 (89)	5 (83)	1.0	26 (67)	11 (85)	.30
Access to care n, (%)	12 (60)	7 (100)	.07	18 (95)	4 (67)	.13	30 (77)	11 (85)	.71
Adverse childhood experiences n, (%)	4 (20)	5 (71)	.02	17 (89)	5 (83)	1.0	21 (54)	8 (62)	.75

**Table 5:** Resident statements of reflection regarding one thing they had done differently in continuity clinic because of the simulation exercise

“Screening patients (usually low weight) for food insecurity”
“Utilized those skills to open the convo about these topics”
“Talking about uncomfortable things like ACEs/food insecurity with ease”
“Write letter to landlord and discuss food insecurity”
“Offered to write letters, let families know that they have a right to ask”
“I feel I remember to ask about food insecurity more often”
“I’ve been more explicit asking about food insecurity, etc.”
“Asking more direct and specific questions regarding housing, transportation, or food insecurity.”
“Know community resources, to be aware of the adversity and to ask about it”
“Asked more directly about [sic]SDoH.”
“Deliberately look at the food insecurity portion of the survey”

## Discussion

Participation in this simulation-based curriculum improved resident comfort with clinical discussions of SDoH. Our curriculum had the greatest effect on PGY-1 participants in each of the four scenarios with the greatest increases noted in those involving housing insecurity and ACEs. Resident participants also reported intentional engagement in conversations regarding SDoH in their continuity clinic practice. Overall, resident feedback on the curriculum was positive and over 80% of participants strongly recommended offering this curriculum to future trainees.

The implementation of this curriculum may be an important intervention for helping pediatric residents meet the professional call to address SDoH in practice [5, 24, 25] and aligns with the National Academies of Sciences, Engineering and Medicine recommended activities of increasing health care awareness on SDoH, using social risk information to inform clinical care decision making, and linking patients to appropriate resources [26]. Numerous screening tools are available to physicians to help identify patients in clinical practice experiencing SDoH regardless of provider competence in doing so [27]. However, using these tools to recognize individuals at-risk of adverse effects from SDoH does not imply a provider can adequately address or even feel comfortable addressing a positive screening result [28]. In our curriculum, participants practiced interpreting and explaining results of similar screening tools, as well as building confidence for carrying out recommended screening activities. Our curriculum focusing on increasing comfort and providing feedback for improvement contributes to efforts to close an important educational gap.

In our study, residents had low baseline levels of comfort addressing many SDoH prior to participating in the curriculum. Our findings add a trainee perspective regarding discomfort with discussing SDoH to previous studies focused on attendings. In a study by Barndige et al., nearly half of pediatric providers reported uncertainty with handling a positive screening result for food insecurity as a significant barrier to performing routine screening [29]. There is evidence that caregivers of children living with food insecurity have preferences about provider communication, including empathic communication, normalization of food insecurity, and the provision of resources [30]. The feedback residents received during the simulations in our curriculum focused on similar content and learning communication strategies that are aligned with caregiver preferences.

The increase in resident comfort in discussing SDoH after participating in this curriculum, as well as the sustained improvement longitudinally, are important educational outcomes. After our intervention, 72% of trainees agreed that they would be comfortable discussing ACEs in a clinical setting, with a greater proportion of PGY-1 residents agreeing after the exercise compared to before (65% vs. 5%; *p* < .01). Accordingly, the optimal period for this training may be during the intern year. Similar trends were observed for the remaining SDoH involving food insecurity, housing insecurity, and limited access to care. We also observed that the increase in comfort was largely sustained during 9–12 months after the initial intervention with the exception of PGY-1 comfort with ACEs. Although this difference failed to reach statistical significance, the magnitude of decrease (-21%) in the proportion of residents reporting comfort with discussing ACEs highlights the need for future studies that focus on the sustainability in educational and behavioral changes associated with a simulation-based curriculum. Nonetheless, our results suggest that, in addition to being well-received, simulation-based experiences can be used to increase resident comfort with discussing SDoH in a clinical setting.

The residents participating in our curriculum reported a relatively low number of baseline number of conversations about SDoH in continuity clinic. Although the number of self-reported discussions about SDoH in clinic did not significantly increase among the overall cohort of residents during the study period, more PGY-1 residents did report having ≥ 2 conversations in continuity clinic at the end of the training year compared to prior to the simulation experience. This adds to existing evidence in undergraduate and graduate medical education that SDoH education increases the frequency of addressing SDoH in a clinical context. Upon completion of a lecture-based SDoH curriculum, pediatric clerkship students improved their overall knowledge regarding SDoH and also reported increased frequency in discussions of food insecurity with patients [8]. Although it is unknown whether these habits continued during residency, this nonetheless highlights the importance of studying the long-term impact of SDoH curricular interventions. In a separate study, PGY-2 and − 3 residents were exposed to a curriculum involving videos with scenarios that should trigger screening for various SDoH. Compared to a control group of residents not receiving the curriculum, more residents in the intervention arm screened for sources of familial support and housing conditions [31]. Similarly, exposure to a video-based curriculum resulted in increased screening for domestic violence and parental depression compared to a control group not receiving the education [32]. The impact of curricula on screening is significant because implementation of systematic approaches to identifying needs related to SDoH have been shown to improve access to resources for families [2]. Institutional process changes may also increase the number of opportunities for conversations about SDoH such as ACEs [16] as well as other related topics including personal benefits, housing, child education, legal and personal safety [33]. However, given barriers to addressing SDoH in practice reported by residents, [31] educational interventions, including those incorporating simulation, either as a supplemental training experience or as the sole curricular component, may be a feasible method to provide additional exposure to SDoH clinical scenarios and help residents more effectively integrate this into their personal clinical practice. Furthermore, the benefit of adaptable simulation-based curricula that allow training programs to address the unique and everchanging needs facing individual institutions and communities may outweigh the lack of malleability in static curricula such as informational videos.

Residents found our curriculum useful for learning about community resources and practicing clinically-oriented conversations related to SDoH as has been described previously [7, 9, 34]. Klein et al. also reported trainees experiencing impactful realizations about family circumstances, reflecting about self-perceptions and practices, and gaining knowledge regarding community-based resources after a two-week curriculum combining didactics and immersive experiences involving poverty and provision of health care to underserved populations [9]. Similarly, internal medicine residents appreciated both the burden of and system-level factors contributing to SDoH while gaining a sense of empowerment to advocate for improved access to resources [7].

The majority of residents expressed that these simulations were an educationally valuable method of teaching SDoH, despite not having an extensive pre-simulation didactic session on the topic. This, along with our other learner outcomes of the curriculum, is significant because little has been described about the impact of simulations focused on discussing SDoH. A recent scoping review of curricula reporting outcomes relating to teaching SDoH [35] identified that only one of 12 studies included a simulation experience. However, the impact of the simulation component was not independently evaluated [36]. A simulation and video training curriculum enhanced residents’ understanding of discussions regarding ACEs with adult patients but did not address the unique family-centered interactions of pediatrics [37]. Our findings extend the support for using simulation training for this topic in pediatrics, where early intervention to mitigate the negative impact of ACEs is still possible.

Our initial study of this curriculum has several limitations. First, our study was limited by its smaller sample size and low response rate for the longitudinal follow-up. However, collection of data across two years do assist with increasing the generalizability of our findings despite the limited sample size and response rates. Furthermore, a larger sample size would likely add statistical power to relevant magnitudes of differences observed in comfort and changes in behavior that are relevant to medical educators but failed to reach statistical significance. Nonetheless, these initial data support our pursuit of future efforts to better understand the role for simulation in SDoH discussions with patients and families. Second, our survey measuring resident self-reported comfort with the scenarios may not necessarily translate to competence with these discussions.Additionally, although our study only measured comfort as opposed to self-efficacy, per Albert Bandura’s social-cognitive theory, comfort is as important as knowledge and skills to perform a task and therefore helpful for performance improvements [38, 39]. Future efforts focused on developing a critical task list that, when combined with direct observation of residents in live clinical encounters, would allow for a more meaningful measurement of behavioral change. Finally, we recognize that there are inherent challenges that may limit the use of simulation as an educational tool including a lack of clinical validity of each scenario and investigator bias towards the educational benefits of simulation [40]. Furthermore, the challenges and sentiments of individuals affected by SDoH are difficult to accurately simulate and are subject to projection of personal biases. We attempted to reduce the effects of these biases by having our scenarios reviewed by other experts in medical education but cannot completely eliminate the possibility for these biases.

In summary, we successfully implemented a SDoH simulation curriculum at our institution and have shown that simulation increased resident comfort with discussing SDH in a clinical setting. Our future efforts are focused on developing critical action checklists for each scenario that will assist residents with identifying actionable areas for improvement. We also aim to investigate if exposure to the simulations and increased comfort level with each scenario correlates with an increased likelihood of engaging in these conversations in a clinical setting.

## Supplementary Information

**Additional file 1: **Retrospective pretest posttest survey used in the study.

## Data Availability

The datasets analyzed during the current study are available from the corresponding author on reasonable request.

## Abbreviations

ACE: adverse childhood experience
PGY: post-graduate year
SP: simulated parent
SDoH: social determinants of health
