# Supplementary material for: Using simulation to increase resident comfort discussing social determinants of health
Source: BMC Med Educ. 2021 Dec 6;21:601. doi: 10.1186/s12909-021-03044-5 (PMC8647375; doi:10.1186/s12909-021-03044-5)
Supplement: Supplementary file 1 — Additional file 1: Retrospective pretest posttest survey used in the study. [file 12909_2021_3044_MOESM1_ESM.docx]

**Using Simulation to Increase Resident Comfort Discussing Social Determinants of Health**

John M Morrison MD PhD^1,2^: 601 Fifth Street South Suite 501Saint Petersburg, FL 33701, jmorri86@jhmi.edu

Sarah M. Marsicek MD^3^ : 601 East Rollins Street Orlando, FL 32803, sarah.marsicek.md@adventhealth.com

Akshata M Hopkins MD^1,2^: 601 Fifth Street South Suite 501Saint Petersburg, FL 33701, ahopki16@jhmi.edu

Robert A Dudas MD^1,2^: 601 Fifth Street South Suite 501Saint Petersburg, FL 33701, rdudas1@jhmi.edu

Kimberly R Collins MD^1,2^: 601 Fifth Street South Suite 501Saint Petersburg, FL 33701, kcolli39@jhmi.edu

Affiliations:

^1^Department of Pediatrics, Johns Hopkins University School of Medicine, Baltimore MD

^2^Division of Pediatric Hospital Medicine, Department of Pediatric Medicine, Johns Hopkins All Children’s Hospital, St Petersburg FL

^3^Division of Pediatric Hospital Medicine, Department of Pediatric Medicine, AdventHealth for Children, Orlando FL

Send all correspondence to:

John M Morrison MD PhD

Division of Pediatric Hospital Medicine, Johns Hopkins All Children’s Hospital

601 Fifth Street South

Suite 501

Saint Petersburg, FL 33701

KEY WORDS: social determinants of health, simulation, graduate medical education

RUNNING TITLE: Simulation and Comfort With Social Determinants of Health

FUNDING: This project was not supported by any funding.

ABSTRACT WORD COUNT: 247/250

MANUSCRIPT WORD COUNT: 3500/3500

DISCLOSURES: All authors have no financial or intellectual conflicts of interest to disclose.

CONTRIBUTIONS:

Dr. Morrison conceptualized and designed the study, collected primary data, drafted the initial manuscript, and approved the final manuscript as submitted.

Drs. Mariscek, Hopkins, Dudas, and Collins collected primary data, reviewed and revised the manuscript, and approved the final manuscript as submitted.

**Supplemental Figure 1.**

**
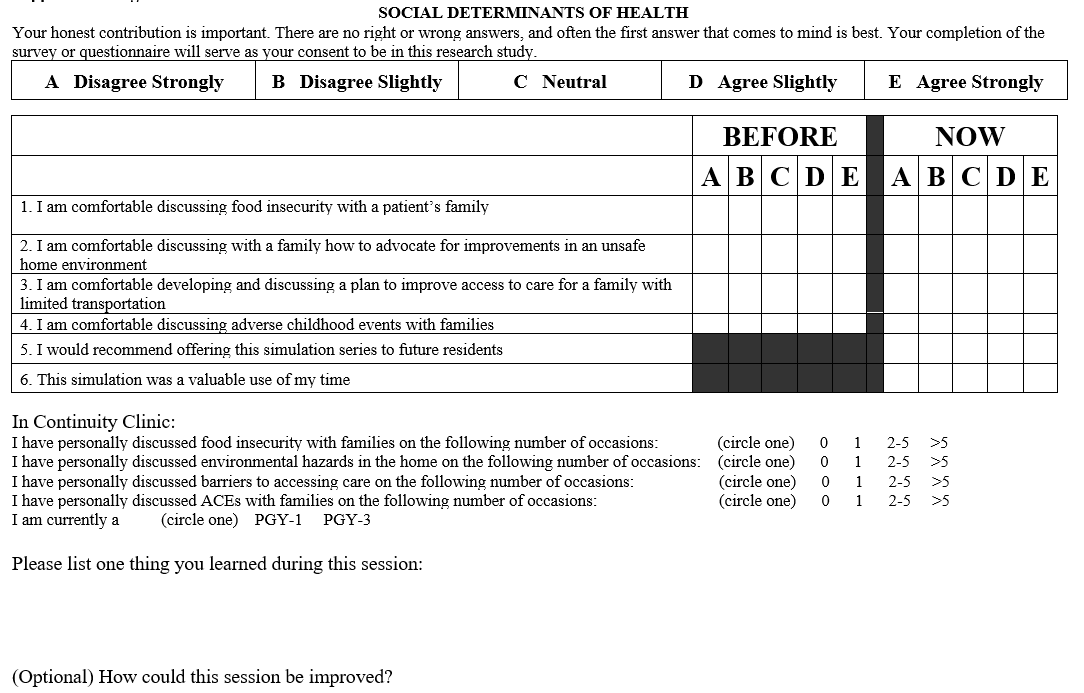
**
